# Changes in work-related stressors before and during the COVID-19 pandemic: differences by gender and parental status

**DOI:** 10.1007/s00420-022-01933-w

**Published:** 2022-11-11

**Authors:** Insa Backhaus, Hanno Hoven, Clare Bambra, Tuula Oksanen, Mariann Rigó, Cristina di Tecco, Sergio Iavicoli, Nico Dragano

**Affiliations:** 1 Institute of Medical Sociology, Centre for Health and Society, Medical Faculty, Heinrich Heine University Duesseldorf, Moorenstrasse 5, 40225 Duesseldorf, Germany; 2 Faculty of Medical Sciences, Population Health Sciences Institute, Newcastle University, Newcastle-Upon-Tyne, UK; 3 Institute of Public Health and Clinical Nutrition, University of Eastern Finland, Kuopio, Finland; 4 Italian Workers’ Compensation Authority (INAIL), Department of Occupational and Environmental Medicine, Epidemiology and Hygiene, Rome, Italy; 5 Directorate for Communication and International Affairs, Ministry of Health, Rome, Italy

**Keywords:** Women, Parents, Work, Stress, Trend analysis, Inequality

## Abstract

**Purpose:**

The COVID-19 pandemic changed people’s working conditions worldwide and research suggests increases in work stressors. However, it is not known to what extent these changes differ by gender or parental status. In the present study, we investigate trends in work stressors and whether these differ by gender and parental status.

**Methods:**

We used cross-sectional time series data of the European Working Conditions Survey of 2015 and Living, Working and COVID-19 survey of spring 2020 to examine trends in work stressors by gender and parental status. Work stressors were working in leisure time, lack of psychological detachment and work–life conflict. We applied three-way multilevel regressions reporting prevalence ratios and reported predicted probabilities and average marginal effects to show trends and differences in changes in work stressors.

**Results:**

Our multilevel regression results showed elevated prevalence ratios during the pandemic for working leisure time (PR: 1.43, 95% CI 1.34–1.53), psychological detachment (PR: 1.70, 95% CI 1.45–1.99) and work–life conflict (PR: 1.29, 95% CI 1.17–1.43) compared to before the pandemic. Except for working in leisure time, the increase was more significant among women and mothers. The proportion of work–life conflict in 2020 was 20.7% (95% CI 18.7–22.9) for men and 25.8% (95% CI 24.0–27.6) for women, equalling a difference of 5.1% (*p* < 0.001).

**Conclusions:**

There is evidence that work stressors increased disproportionately for women and mothers. This needs to be monitored and addressed to prevent widening gender inequalities in the quality of work.

**Supplementary Information:**

The online version contains supplementary material available at 10.1007/s00420-022-01933-w.

## Introduction

Globalisation and digitalisation are affecting psychosocial working conditions across Europe and there is uncertainty whether they are changing for the better or worse. While some studies suggest that psychosocial working conditions have deteriorated over the past decades (Rigó et al. 2021; Myers et al. 2019; Malard et al. 2015), other studies have not found a uniform trend (Malard et al. 2013; LaMontagne et al. 2013). There is also uncertainty about whether trends in psychosocial working conditions are equally distributed among workers. Studies are currently scarce and the few studies assessing inequalities are country specific or focus on differential changes according to occupational status (e.g., lower versus higher skilled workers) (Malard et al. 2015; Rigó et al. 2021), but neglect a prevalent problem in labour markets—gender inequality.

Gender inequalities in the labour market, however, are a persistent problem and there is a growing concern of international organisations, such as the International Labour Organization and UN Women, that the COVID-19 pandemic has shifted working conditions for women negatively, diminished gender equality and may even have exacerbated gender inequalities (International Labour Organization 2021a, b; International Labour Organization and UN Women 2021b). Early pandemic data shows that women experienced a significantly greater job and income loss and were more likely to experience work–life conflict (International Labour Organization 2021a, b; Möhring et al. 2020; Zoch et al. 2021). Specifically, many women were forced to increase their amount of unpaid care work (Xue and McMunn 2021; Zoch et al. 2021) and to decrease their paid working hours (Xue and McMunn 2021; Matteazzi and Scherer 2021; Collins et al. 2020) as a result of nationwide lockdowns and the closure of childcare facilities. The additional time spent in unpaid care work may have limited women’s ability to keep their jobs and could have triggered additional stress and mental illness for them (Beauregard et al. 2018; Adisa et al. 2021). However, comparative analyses using pre-pandemic and pandemic data confirming rising inequalities are currently lacking.

Something that is well-known is that psychosocial work stressors (i.e. working conditions) can be detrimental to worker’s well-being and health (Jerg-Bretzke et al. 2020; Frone et al. 1996; Leineweber et al. 2013; Hämmig et al. 2009). There is a very sizeable amount of literature linking psychosocial work stressors with various stress-related physical (e.g. headaches) and mental health conditions (e.g. depressive symptoms). Job strain, for example, may lead to coronary heart disease (Kivimäki et al. 2012; Sara et al. 2018), an imbalance between work and private life can cause emotional exhaustion and problematic alcohol consumption (Leineweber et al. 2013), and work–life conflict has been linked to fatigue and back- and headaches (Hämmig et al. 2009).

Given the unfavourable outcomes, it is important to understand who is most affected by work-related stressors and to possibly identify patterns of inequalities. Therefore, we aim in the present study to investigate changes in work-related stressors by gender and parenthood using time series data for the 27 EU countries. Specifically, we explore differences between female and male workers and those living with a young child. We hypothesized that work-related stressors will have increased for all workers, but that the increase will be greater among female workers and particularly those living with at least one child under the age of 18 years. To assess changes in work-related stressors, we applied a comparative multilevel approach using data from Eurofound’s latest pre-pandemic European Working Conditions Survey (EWCS) of 2015 and Eurofound’s COVID-19 pandemic-related survey on Living, Working and COVID-19 (COVID-19S). These allowed us to observe trends in the psychosocial work environment in European countries in a multilevel framework (workers nested within country-years, nested within countries). We focused on three critical work-related stressors potentially affected by the COVID-19 pandemic, namely: a) working in leisure time, b) lack of psychological detachment and c) work–life conflict.

## Methods

### Data

In the present study, we used data for the 27 EU member states from Eurofound’s sixth wave of the EWCS and data from Eurofound’s first wave of the COVID-19S (Eurofound 2015, 2020). The EWCS is a cross-sectional survey that has been carried out every 5 years by Eurofound in 35 European countries since 1990. It collects data on the working conditions and demographic characteristics of the European working population (i.e. only people in employment). The COVID-19S was launched by Eurofound in April 2020 and provides detailed information on living and working conditions during the COVID-19 pandemic among the general population of the 27 EU member states. A detailed description of the survey’s methodology can be found in Eurofound’s methodological reports (Eurofound 2015, 2020). While the reference period in the questionnaires is somewhat different, the past 12 months in the EWCS vs. the past 2 weeks in the COVID-19S, the EWCS 2015 and the first wave (Spring 2020) of the COVID-19S are highly comparable.

### Study sample

The combined sample of the 2015 wave of the EWCS and the COVID-19S consists of 111,996 respondents (2015: *n* = 43,850, response rate: 43% and 2020: *n* = 68,146, response rate: n/a) from 35 European countries: 27 EU Member States and Albania, Macedonia, Montenegro, Norway, Serbia, Switzerland, Turkey and the United Kingdom. We omitted non-EU countries from the analysis as they did not participate in both surveys, leading to *n* = 102,288 (91.3% of the initial sample). We also excluded participants under 18 years or over 67 years (*n* = 6,481) (i.e. EWCS and COVID-19S) and unemployed individuals (*n* = 18,571; i.e. COVID-19S). These cut-offs were chosen because in many EU countries, the age of majority is 18 and the general retirement age is 67 years. Finally, we excluded non-binary participants (*n* = 206) and those with missing data (*n* = 3,741), resulting in an analytic sample of 73,296 individuals (EWCS: *n* = 31,401; COVID-19S: *n* = 41,895; 65% of the total initial sample).

### Variables and measures

#### Work-related stressors

In this study, we focused on three work-related stressors that were available in both the EWCS and the COVID-19S. These included: a) working in leisure time, b) lack of psychological detachment (i.e. inability to detach from work) and c) work–life conflict (see Supplementary Table S1 for the specific survey items).

Working in leisure: working in leisure time was determined by asking participants, “Over the last 2 weeks, how often have you worked in your free time to meet work demands?” The answering options included: “every day”, “every other day”, “once or twice a week”, “less often” and “never”. We categorized the responses for our analyses into “less often/never” and “once or twice a week/every day”, the latter representing adverse working conditions.

Psychological detachment: psychological detachment from work refers to a state where people mentally disconnect from work and do not think about their job-related activities during their leisure time (Sonnentag 2012). Lack of psychological detachment was based on a single question inquiring, “How often have you kept worrying about work when you were not working”. Response options were recoded into a dichotomous variable (sometimes/always and rarely/never).

Work–life conflict: work–life conflict can be understood as tensions arising when combining work and private life. Scholars have specifically described work–life conflict as a form of inter-role conflict that arises when one’s behavioural demands of the work role conflict with those of the family role (Kossek and Lee 2017). A variety of indicators have been used previously to measure work–life, either individually or in combination as a composite index. Following the framework of resources and demands suggested by Voydanoff (2005), the combination of two questions in the EWCS and COVID-19S covered conflict originating in the workplace and affecting the non-work domain. The questions were “How often have you: (a) Felt too tired after work to do some of the household jobs which needed to be done, and (b) Found that your job prevented you from giving the time you wanted to your family”. Answering options ranged from “never” to “always”, which were scored with the values 0 to 4. In the present study, we created a composite index of these two questions with a minimum of 0 and a maximum of 8 points. Regarding the analyses, we dichotomised a score with 0 to 4 points indicating ‘no work-life conflict’ and a score of 5 points and above indicating ‘work-life conflict’. A sensitivity analysis showed that a cut-off between 6 and 7 points does not produce a significant change of results. A similar approach was applied by Borgmann et al. (2019).

#### Covariates

Gender: gender was ascertained in the COVID-19S by asking participants “How would you describe yourself?”. Answering options were “female”, “male” or “in another way”. Gender was ascertained in the EWCS through the interviewer, and answering options included “female”, “male” and “don’t know”. We excluded participants for the analyses with a non-binary gender (*n* = 206).

Parental status: we constructed four groups regarding parental status: female living with a child under the age of 18, female not living with a child under the age of 18, male living with a child under the age of 18 and male not living with a child under the age of 18. We refer to the first and third groups as mothers and fathers for the ease of interpretation and clear distinction in the text, tables and figures. We only included children aged 18 years or younger because in many European countries, those who have reached 18 are considered legal adults and can, thus, live their lives accordingly.

Possible confounding factors: we included age (18–35, 36–55 and ≥ 56 years), education (low, medium and high) and household characteristics (i.e. marital/partner status: living in the same household as spouse or partner) to control for possible confounding. Education was assessed in both the EWCS and COVID-19S according to the 2011 International Standard Classification of Education and was regrouped into low (i.e. pre-primary, primary or lower secondary education), medium (i.e. upper secondary or post-secondary education) and high (i.e. first and second stage of tertiary education) education (UNESCO 2013). Age was ascertained in both surveys by asking respondents “how old are you?”. In the COVID-19S, living in the same household as one’s spouse/partner was assessed by asking “Do you have a spouse/partner who lives in your household?”, with answering options “Yes” or “No”, whereas the spouse/partner status was ascertained in the EWCS by asking respondents to indicate the relationship to their household member.

### Statistical analyses

We first compared changes in working conditions across the different survey years using descriptive statistics to address whether health-related work stressors had changed between 2015 and 2020. We then, following the suggestion by Fairbrother (2014), regressed working conditions on a set of covariates applying three-way multilevel Poisson regression analysis for comparative longitudinal data sets and binary data with workers (level 1) nested within country-years (level 2) and within countries (level 3), incorporating a random intercept for countries and country-years in addition to the individual error term. We use three-level Poisson regressions for binary outcomes as they allow the estimation of prevalence ratios (PRs). These are more intuitive to interpret compared to odds ratios (Knol et al. 2012). We adjusted the three-level multilevel Poisson regression models for age (18–35, 36–55 and ≥ 56 years), education (low, medium and high) and marital/partner status (living with partner and not living with partner). We assessed the change in working conditions between 2015 and 2020 separately by gender and parental status by including interaction terms between these covariates and the wave dummy. Specifically, we computed predicted probabilities for adverse work stressors for each gender and parental status by wave. The changes in predicted probabilities between the waves are expressed by average marginal effects (AMEs) to facilitate interpretation. Moreover, to simplify the interpretation of the results we used binary outcomes for all analyses. All calculations were carried out using Stata 15 (Stata Corporation, College Station, Texas, USA). Regarding the descriptive analyses, we used weights provided by Eurofound to produce numbers that are representative of EU27 averages. Concerning the regression analysis, we did not use weights as data as country differences in terms of composition and data representativeness are taken into account by a wide range of explanatory variables.

## Results

Table 1 gives an overview of the sample characteristics and the prevalence of work stressors in 2015 and 2020. In 2020, 47% of respondents were female, 53% were between the ages 36 and 55 years and 34% had a least one child under the age of 18 years. The proportion of employees working in their free time, experiencing a lack of psychological detachment and work–life conflict increased over time from 22 to 34%, from 15 to 29% and from 17 to 22% respectively (Table 1).

**Table 1:** Sample characteristics and description of psychosocial working conditions (*n* = 73,296)

	2015 (*n* = 31,401) *n* (Col. %^a^)	2020 (*n* = 41,895) *n* (Col. %^a^)
Gender		
Female	16,156 (52.5)	29,502 (47.4)
Male	15,245 (47.5)	12,393 (52.6)
Age		
18–35 years	9049 (29.9)	8950 (28.4)
36–55 years	17,076 (54.4)	23,752 (52.8)
56 years and older	5276 (15.7)	9193 (18.8)
Marital/partner status		
Living with partner	20,433 (69.7)	28,630 (66.8)
Not living with partner	10,846 (30.3)	12,450 (33.2)
Education		
Low	1133 (3.3)	916 (6.5)
Medium	19,826 (62.9)	11,067 (59.3)
High	10,441 (33.8)	29,912 (34.2)
Living with an under 18-year-old		
Yes	10,350 (34.6)	14,958 (34.4)
No	21,051(65.4)	26,910 (65.6)
Worked in leisure time		
Less often/Never	24,557 (78.0)	24,508 (66.1)
Once twice a week/Every day	6844 (22.0)	17,387 (33.9)
Lack of psychological detachment		
Rarely / Never	26,555 (84.7)	30,154 (70.5)
Sometimes/Always	4856 (15.3)	11,742 (29.5)
Work-life conflict		
Rarely/Never	25,928 (82.7)	31,739 (77.8)
Sometimes/Always	5473 (17.3)	10,156 (22.2)

^a^Weighted percentages

### Work-related stressors

Table 2 shows the results of three-level multilevel Poisson regression models adjusted for covariates and based on the sample of EU27 countries. The results show that the prevalence of reporting working in leisure time (PR: 1.43, 95% CI 1.34–1.53), lack of psychological detachment (PR: 1.70, 95% CI 1.45–1.99) and work–life conflict (PR: 1.29; 95% CI 1.17–1.43) was higher in 2020 compared to 2015. The results also indicate that women were less likely to detach from work than men (PR: 1.01, 95% CI 1.00–1.06) and more likely to experience work–life conflict (PR 1.10; 95% CI 1.03–1.17) compared to male workers. Moreover, the PR that parents, both mothers and fathers, were generally more likely to experience work–life conflict when compared to employees without children. However, the PR for work–life conflict was particularly elevated among mothers (PR: 1.37, 95% CI 1.31–1.45), while the PR for a lack of psychological detachment was more pronounced among fathers (PR: 1.14, 95% CI 1.05–1.23). Somewhat similar results were found for working in leisure time, with fathers having a PR of 1.22 (95% CI 1.13–1.32) and mothers of 0.96 (95% CI 0.89–1.02) when compared to employees without children.

**Table 2:** Prevalence Ratios (PRs) for experiencing adverse psychosocial working conditions by survey year and gender

	Working in leisure time	Lack of psychological detachment	Work-life conflict
By gender	PR (95% CI)	PR (95% CI)	PR (95% CI)
Survey Year			
2015	Ref	Ref	Ref
2020	1.43 (1.34–1.53)	1.70 (1.45–1.99)	1.29 (1.17–1.43)
Gender			
Male	Ref	Ref	Ref
Female	0.81 (0.76–0.86)	1.01 (1.00–1.06)	1.10 (1.03–1.17)
By parental Status			
Survey Year			
2015	Ref	Ref	Ref
2020	1.49 (1.37–1.61)	1.79 (1.51–2.12)	1.39 (1.27–1.53)
Parental status			
Not a parent	Ref	Ref	
Mother	0.96 (0.89–1.02)	1.05 (1.02–1.12)	1.37 (1.31–1.45)
Father	1.22 (1.13–1.31)	1.14 (1.05–1.23)	1.29 (1.21–1.38)
*N*	73,296	73,296	73,296
Groups	27	27	27

Estimates are based on multilevel regressions analysing the association between covariates and psychosocial working conditions with three levels (level 1: individual, level 2: country-years, level 3: country). Adjusted for age, education, marital/partner status

95% *CI* = 95% confidence interval

### Trends in work-related stressors by gender

**Fig. 1:**
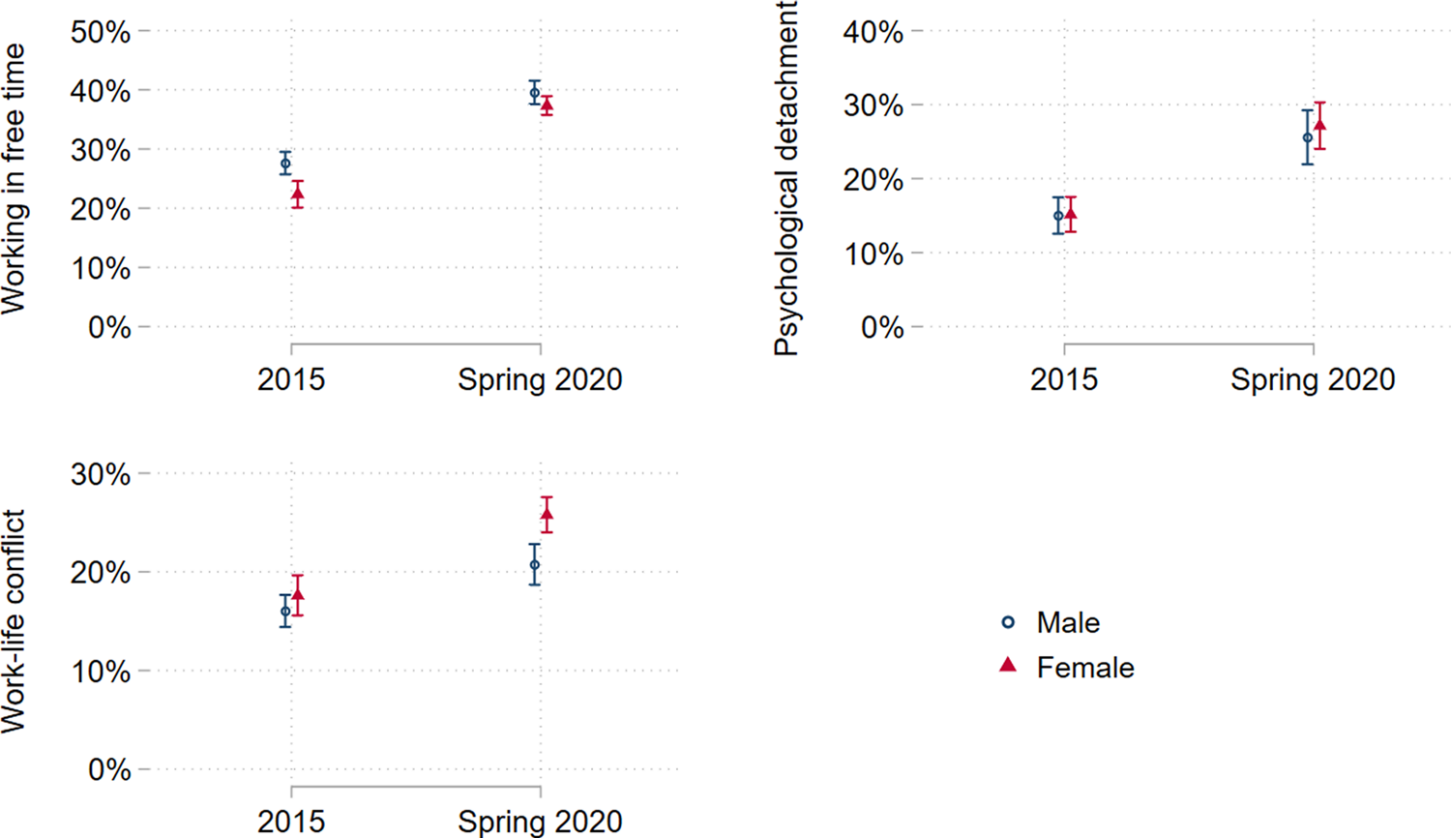
Predicted probabilities of work stressors by gender. Predicted probabilities and their 95% confidence intervals. Computation based on three-level multilevel regressions as specified in Table 3, adjusted for age, education and marital/partner status. Error bars represent 95% confidence intervals

Table 3 and Fig. 1 show a significant increase in all psychosocial work conditions examined among both female and male workers from 2015 to 2020. However, changes were not uniformly distributed and a significantly larger increase for female workers was noticed in the domains lack of psychological detachment and work–life conflict from 2015 to 2020. Looking at work–life conflict, for instance, one can notice that the predicted probability of work–life conflict for male workers was 16.0% (95% CI 14.4–17.7) and 17.6% (95% CI 15.6–19.6) for female workers, showing a difference of 1.6% (*p* = 0.005) before the COVID-19 pandemic (2015). Those numbers rose with the onset of the COVID-19 pandemic to 20.7% (95% CI 18.7–22.8) and 25.8% (95% CI 24.0–27.6), respectively, resulting in a difference of 5.1% (*p* < 0.001). A similar observation can be made for a lack of psychological detachment. There were no significant differences in the predicted probability of a lack of psychological detachment for male workers (15.0%; 95% CI 12.6–17.5) and female workers (15.1%, 95% CI 12.8–17.5) in 2015, however, this changed in 2020. The predicted probability of a lack of psychological detachment during the COVID-19 pandemic was 25.6% (95% CI 21.9–29.3) for male workers, but 27.2% (95% CI 24.0–30.3) for female workers, producing a difference of 1.6% (*p* = 0.050). Furthermore, though an increase in a lack of psychological detachment and work–life conflict is noticeable for both female and male workers, the increase is significantly steeper among female workers. This pattern is illustrated in the bottom right-hand corner of Table 3, which shows the value of the difference in trends (from 2015 to 2020) between the male and female workers and the corresponding *p*-value. As an illustration, the difference is 3.5% (*p* < 0.001) in the case of work–life conflict. This indicates that the deterioration of work–life conflict from 2015 to 2020 was more pronounced among female workers than male workers.

**Table 3:** Predicted probabilities of psychosocial working conditions by gender, *n* = 73,296

	2015 Predicted Probability (95% CI)	2020 Predicted Probability (95% CI)	AME 2020 vs. 2015 (*p* value)
Working in leisure time			
Female	22.3 (20.1–24.6)	37.3 (34.5–38.9)	15.0 (*p* < 0.001)
Male	27.6 (25.7–29.5)	39.5 (37.5–41.5)	11.9 (*p* < 0.001)
AME Female vs. Male (*p* value)	−5.3 (*p* < 0.001)	−2.2 (*p* = 0.004)	3.1 (*p* = 0.007)
Lack of psychological detachment			
Female	15.1 (12.8–17.5)	27.2 (24.0–30.3)	12.1 (p < 0.001)
Male	15.0 (12.6–17.5)	25.6 (21.9–29.3)	10.6 (*p* < 0.001)
AME Female vs. Male (*p*-value)	0.1 (*p* = 0.753)	1.6 (*p* = 0.050)	1.5 (*p* = 0.046)
Work-life conflict			
Female	17.6 (15.6–19.6)	25.8 (24.0–27.6)	8.2 (*p* < 0.001)
Male	16.0 (14.4–17.7)	20.7 (18.7–22.8)	4.7 (*p* < 0.001)
AME Female vs. Male (*p*-value)	1.6 (*p* = 0.005)	5.1 (*p* < 0.001)	3.5 (*p* < 0.001)

Estimates are based on multilevel regressions on the association between covariates and psychosocial working conditions with three levels (level 1: individual, level 2: country-years, level 3: country), adjusted for age, education and marital/partner status

95% *CI* 95% confidence interval, *AME* average marginal effects

### Trends in work-related stressors by parental status

**Fig. 2:**
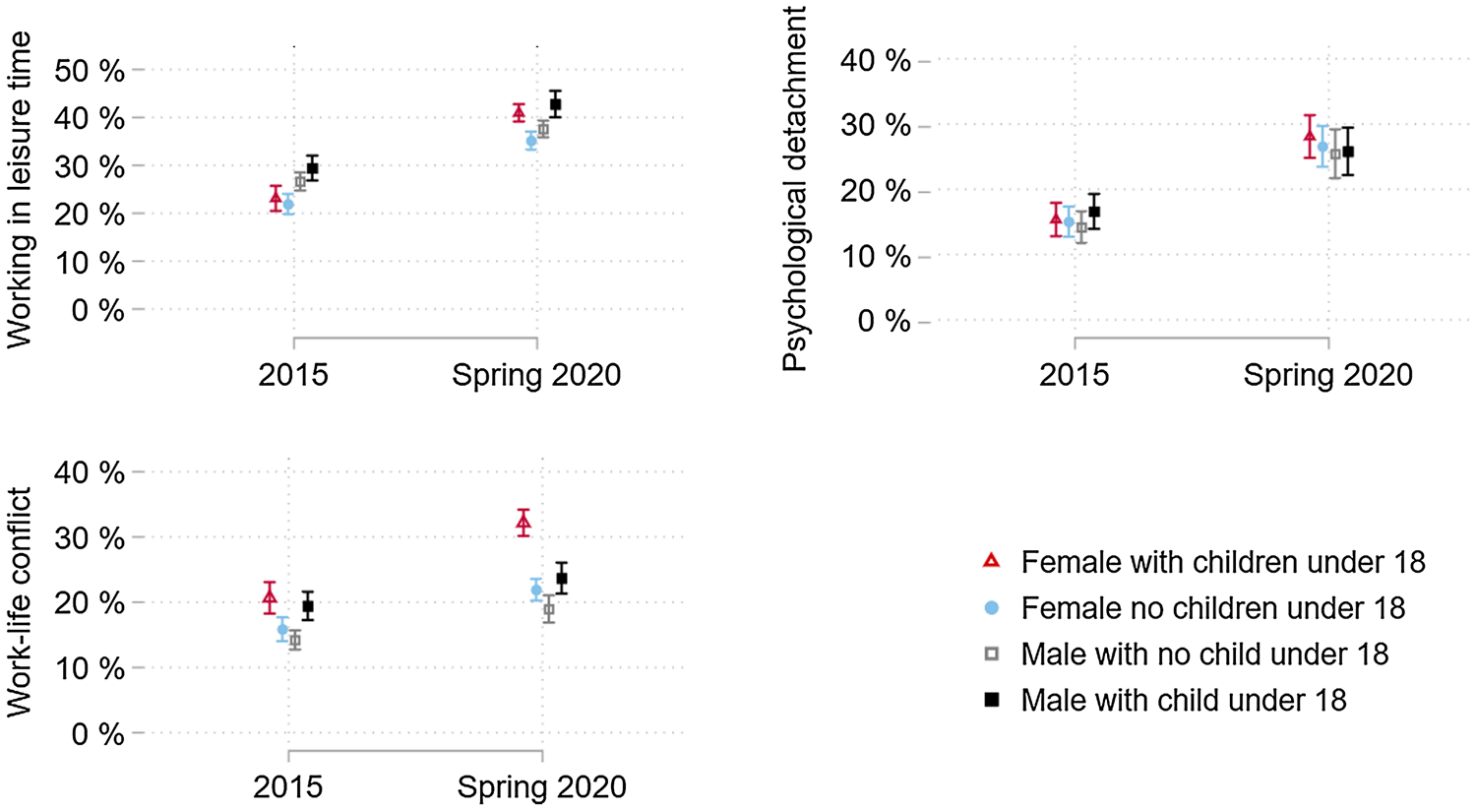
Predicted probabilities of work stressors by gender and parental status. Predicted probabilities and their 95% confidence intervals. Computation based on three-level multilevel regressions as specified in Table 4, adjusted for age, education and marital/partner status. Error bars represent 95% confidence intervals

According to Table 4 and Fig. 2, the predicted probabilities of experiencing work-related stressors differ by the four combinations of gender and parental status. Working in leisure time, lack of psychological detachment and work–life conflict increased across the groups from 2015 to 2020. The increase in a lack of psychological detachment and work–life conflict, however, was more pronounced among mothers living with young children. Exemplarily, work–life conflict among mothers in 2020 was predicted to be 32.2% (95% CI 30.2–34.2), while it was 23.7% (95% CI 21.3–26.0) for fathers, showing a difference of 8.5% (*p* > 0.001). Furthermore, the increase in work–life conflict from 2015 to 2020 is significantly greater among mothers than fathers (right-hand corner of Table 4, AME 2020 vs. 2015). The difference in the AMEs is 7.2% (*p* < 0.001). A similar observation was made for a lack of psychological detachment. In 2020, the lack of psychological detachment among mothers was predicted to be 28.1% (95% CI 24.8–31.4), while it was 25.8% (95% CI 22.1–29.4) among fathers, resulting in a difference of 2.3% (*p* = 0.009). Again, the increase in a lack of psychological detachment was significantly larger among mothers than fathers, which is shown by the AMEs of 3.5% (*p* < 0.001) in the right-hand corner of Table 4 (AME 2020 vs. 2015). In summary, mothers have generally higher predicted probabilities of work-related stressors over time compared to fathers and workers without young children.

**Table 4:** Predicted probabilities of psychosocial working conditions by gender and parental status, *n* = 73,296

	2015 Predicted probability (95% CI)	2020 Predicted probability (95% CI)	AME 2020 vs. 2015 (*p* value)
Working in leisure time			
Mother of a young child	23.1 (20.4–25.7)	40.9 (39.1–42.7)	17.8 (*p* < 0.001)
Father of a young child	29.4 (26.8–32.0)	42.8 (40.0–45.5)	13.4 (*p* < 0.001)
Female with no child under 18	21.9 (19.8–24.0)	35.2 (33.3–37.1)	13.3 (*p* < 0.001)
Male with no child under 18	26.6 (24.7–28.5)	37.6 (35.8–39.3)	11.0 (*p* < 0.001)
AME Mother vs. Father (*p* value)	−6.3 (*p* < 0.001)	−1.9 (*p* = 0.115)	4.4 (*p* = 0.004)
Lack of psychological detachment			
Mother of a young child	15.4 (12.8–17.9)	28.1 (24.8–31.4)	12.7 (*p* < 0.001)
Father of a young child	16.6 (13.9–19.3)	25.8 (22.1–29.4)	9.2 (*p* < 0.001)
Female with no child under 18	15.0 (12.7–17.3)	26.6 (23.5–29.7)	11.6 (*p* < 0.001)
Male with no child under 18	14.2 (11.8–16.6)	25.4 (21.7–29.2)	11.2 (*p* < 0.001)
AME Mother vs. Father (*p* value)	−1.2 (*p* = 0.010)	2.3 (*p* = 0.009)	3.5 (*p* = 0.001)
Work-life conflict			
Mother of a young child	20.7 (18.3–23.1)	32.2 (30.2–34.2)	11.5 (*p* < 0.001)
Father of a young child	19.4 (17.2–21.6)	23.7 (21.3–26.0)	4.3 (*p* < 0.001)
Female with no child under 18	15.9 (14.0–17.7)	21.9 (20.2–23.6)	6.0 (*p* < 0.001)
Male with no child under 18	14.2 (12.7–15.7)	19.0 (16.9–21.1)	4.8 (*p* < 0.001)
AME Mother vs. Father (*p* value)	1.3 (*p* = 0.136)	8.5 (*p* < 0.001)	7.2 (*p* < 0.001)

Estimates are based on multilevel regressions on the association between covariates and psychosocial working conditions with three levels (level 1: individual, level 2: country-years, level 3: country), adjusted for age, education and marital/partner status

95% *CI* = 95% confidence interval, *AME* average marginal effects

## Discussion

The COVID-19 pandemic and the national policy responses, such as lockdowns and working from home rules, had far-reaching implications not just for health but also for the economy, businesses and people’s daily lives worldwide. The present study provides first findings of changes in health-related work stressors in Europe before and during the COVID-19 pandemic and shows differences in changes by gender and parental status.

### Main findings

The findings suggest that psychosocial working conditions deteriorated between 2015 and 2020 for all workers. As such, we observed that working in free time increased by 12%, experiencing a lack of psychological detachment increased by 14% and work–life conflict increased by 5%. Furthermore, when we compared trends by gender and parental status, we found that female workers and those without young children experienced greater increases. Regarding work–life conflict, for instance, we observed an increase of 8% amongst female employees, but only a 5% increase amongst male employees.

### Plausibility of the findings

The findings observed are possibly due to the following reasons. Firstly, with the onset of the COVID-19 pandemic workers across the world faced extensive changes at the workplace. These included—among others—remote working, a change in working hours, and sometimes even job and income loss (Xue and McMunn 2021; Zoch et al. 2021; Graham et al. 2021). Furthermore, as the COVID-19 pandemic progressed, especially fathers and mothers were suddenly confronted with the closure of their children’s care facilities and many had to organize their children’s remote schooling. As a consequence many parents reported increasing conflicts when trying to reconcile work and private life (Reimann et al. 2021). Especially women and mothers reported a double or even a triple burden of being an employee, parent and educator at the same time. Although men and women might be expected to take on equal roles in the family context and men’s contribution to childcare has increased, childcare duties continue to fall disproportionately on mothers, and even more so during the COVID-19 pandemic (Zoch et al. 2021; Adams-Prassl et al. 2020; Fodor et al. 2021). This might especially explain our observed increase in work–life conflict among women and mothers. Specifically, evidence from Europe and the United States suggests that women have taken on more childcare responsibilities during the COVID-19 pandemic than men (Adams-Prassl et al. 2020; Fodor et al. 2021). Fodor and colleagues (Fodor et al. 2021), for instance, found that while men’s contribution to childcare increased in relative terms, in absolute terms, women’s contributions grew significantly more than men’s during the pandemic. Consequently, many women were required to cut down on their paid working hours (Collins et al. 2020). This is important, as a reduction in working hours will probably contribute to the already existing motherhood wage penalty that has been determined in several pre-pandemic studies (Anderson et al. 2003; Budig and Hodges 2010). Secondly, the labour market continues to be gender-segregated and the nature of work is gender-specific (Kreimer 2004). Women tend to cluster in occupations such as healthcare, social work and hospitality, all of which were severely hit by the COVID-19 pandemic (OECD 2021, 2020). In the healthcare sector, for instance, workers suddenly found themselves working in a high-risk profession due to increased exposure to COVID-19 and being exposed to prolonged working hours (Hoedl et al. 2021). Unfortunately, we were unable to control for the employment sector in the present analysis, which would have been important as it may have confounded the relationship between gender and work-related stressors (Kreimer 2004; Kjellsson 2021). Nonetheless, when interpreting the results, one must take into account that the data from 2020 refer to a complex moment of time and of the pandemic. In particular, the data are from spring 2020 when COVID-19 prevention and control measures restrictions were strict, uncertainty high among the population and profound life changes took place.

### Strengths and limitations

Our study has some limitations. A primary limitation is that prevalence rates are based on survey data and varying data quality may lead to over- or under-estimations. For instance, while both the EWCS and the COVID-19 survey are conducted by Eurofound, Eurofound applied a different sampling procedure in the COVID-19 survey than in their regular EWCS. Thus, results of the trend analysis should be interpreted with caution. Moreover, some variables of interest and other potential confounders could not be included in the analysis. Exemplarily, data on an employees’ socio-economic position, type of contract and employment sector (e.g. healthcare), which may have confounded the association between gender and the work-related stressors, were not available. In this sense, we especially acknowledge the importance of the employment sector as it would have been important to control for female- and male-dominated sectors. It would also have been especially preferable to control for the healthcare sector, which is known for its gender segregation and was characterised by prolonged working hours and extreme overtime work for many workers during the pandemic. Additionally, no information on childcare arrangements were available. Furthermore, only two measurement points could be included in the present analyses (i.e. 2015 and 2020) as earlier waves of the EWCS (e.g. 2010) did not include our variables of interest. It is also possible that temporal dynamics of the pandemic, such as a country’s lockdown, and prevention and control measures, affected the working conditions and may have inflated the prevalence rates. The restrictions in northern Europe were rather lenient compared to other European countries where curfews were enacted. However, it was out of the scope of the present study to investigate the effects of a country’s specific lockdown measures on working conditions. Nonetheless, our results give rise to further research relating to the question of how a country’s pandemic-related restrictions and preventions have affected working conditions. Despite these limitations, the study has important strengths. Firstly, the study provides first findings of changes in psychosocial working conditions at the start of the COVID-19 pandemic. It examines a unique period as it covers the time before and during the COVID-19 pandemic, a time when changes at the workplace were intensive. Secondly, this is one of the first studies with a rigorous statistical analysis conducting a cross-country time series analysis using well-established measures of health-related work stressors in the first COVID-19 pandemic wave. Thirdly, the findings of this study provide early evidence of trends in health-related working conditions and shed light on the gender disparities arising.

### Implications for future research

The findings of the present study are important as a vast body of research has consistently shown that poor employment conditions and work-related stressors, such as those examined here, can be a source of poor health (e.g. increased rates of cardiovascular disease, depressive disorders, metabolic syndrome, obesity, risky health behaviours) (Hoven et al. 2021; Madsen et al. 2017; Kouvonen et al. 2016). Furthermore, work–life conflict among women and mothers has been specifically linked to adverse health outcomes, such as worse mental health (Borgmann et al. 2019; Guille et al. 2017). Thus, it is possible that women and mothers will be at particular risk of experiencing adverse health effects due to worsening work-related stressors, possibly even further widening inequalities. These adverse working conditions might also increase the labour market exit for women. Thus, future studies should investigate the impact of changing work-related stressors on inequalities in the labour market and health. In addition, previous studies have suggested that gender inequalities in working conditions can be a result of differences of national policies and welfare-state regimes (Artazcoz et al. 2021, 2016; Korpi et al. 2013). As such, studies found a gap between welfare state models in the north and south of Europe and smaller gender gaps in Nordic countries, which are characterised by a dual career family model and statutory support for work–family reconciliation and higher levels of spending on family support. Although the findings of this study provide early evidence of gender differences in trends in health-related working conditions, well-designed social and employment policies that tackle gendered risks continue to be of great importance. Studies in this area are therefore needed.

## Conclusion

The present study provides first evidence for a deterioration in health-related work stressors between 2015 and 2020. While both women and men experienced an increase in work-related stressors, it seems that this increase is more pronounced among women and specifically amongst mothers. Given the rising gender inequalities, further research on specific mechanisms that may lead to a deterioration in work-related stressors and their effect on health is important to develop political action and policy solutions that promote gender equity.

## Supplementary Information

Below is the link to the electronic supplementary material.

Supplementary file1 (DOCX 15 KB)

## Data Availability

The EWCS datasets are stored at the UK Data Service in Essex, UK, and are publicly available via their website (https://ukdataservice.ac.uk/). Users are required to be registered with the UK Data Service. Users who register have to accept the End User License, which is agreed to during the registration process. Restrictions apply to the availability of the data associated with Eurofound’s “Living, Working and COVID-19 survey”. The data were used under licence for the current study, and are not publicly available. Researchers interested may contact Eurofound directly.
