# Supplementary material for: Changes in work-related stressors before and during the COVID-19 pandemic: differences by gender and parental status
Source: Int Arch Occup Environ Health. 2022 Nov 11;96(3):421–31. doi: 10.1007/s00420-022-01933-w (PMC9651091; doi:10.1007/s00420-022-01933-w)
Supplement: Supplementary file 1 — Supplementary file1 (DOCX 15 KB) [file 420_2022_1933_MOESM1_ESM.docx]

**Supplemental material**

*Table S1: List of EWCS and COVID19S items*

| **Work stress measures** | **EWCS and COVID-19S items** | **Response categories** |
| --- | --- | --- |
| Working in leisure time | Over the last 2 weeks/12 months, how often have you worked in your free time to meet work demands? | 1 (Daily)  5 (Never) |
| Lack of psychological detachment | How often (in the last 2 weeks/last 12 months) have you kept worrying about work when you were not working? | 1 (Always)  5 (Never) |
| Work-life conflict | How often (in the last 2 weeks/last 12 months) have you:  a) Felt too tired after work to do some of the household jobs which need to be done, and  b) Found that your job prevented you from giving the time you wanted to your family | 1 (Always)  5 (Never) |

Figure S1: Flow diagram of sample selection

| EWCS (2015)  n = 43,850 | COVID-19 Survey  n = 68,146 |  |  |
| --- | --- | --- | --- |
| ↓ | |  |  |
| Combined sample  n = 111,996 | |  |  |
| ↓ | | → | Exclusion of non-EU countries  n = 9,708 |
| Combined sample without non-EU countries  n = 102,288 | |  |  |
| ↓ | | → | Exclusion of:  non-bainary participants (n=206)  <18 years, >67 years (n=6,481)  unemployed individuals (n=18,571)  n = 25,258 |
| Combined sample without non-EU countries  n= 77,030 | |  |  |
| ↓ | | → | Exclusion of:  Missing data  n = 3,741 |
| Final analytic sample  n = 73,289 | |  |  |
| ↓ | ↓ |  |  |
| EWCS (2015)  n = 31,401 | COVID-19 survey  n = 41,895 |  |  |
